# Decreased expression of Yes-associated protein is associated with outcome in the luminal A breast cancer subgroup and with an impaired tamoxifen response

**DOI:** 10.1186/1471-2407-14-119

**Published:** 2014-02-22

**Authors:** Sophie Lehn, Nicholas P Tobin, Andrew H Sims, Olle Stål, Karin Jirström, Håkan Axelson, Göran Landberg

**Affiliations:** 1 Center for Molecular Pathology, Department of Laboratory Medicine, Lund University, Skåne University Hospital, 205 02 Malmö, Sweden; 2 Cancer Center Karolinska, Karolinska Institutet and University Hospital, 171 76 Stockholm, Sweden; 3 Applied Bioinformatics of Cancer, University of Edinburgh Cancer Research Centre, Carrington Crescent, Edinburgh EH4 2XR, UK; 4 Division of Oncology, Department of Clinical and Experimental Medicine, Faculty of Health Sciences, Linköping University, Linköping, Sweden; 5 Department of Clinical Sciences, Division of Pathology, Lund University, Skåne University Hospital, 221 85 Lund, Sweden; 6 Translational Cancer Research, Department of Laboratory Medicine, Lund University, Medicon Village building 404A3, 223 81 Lund, Sweden; 7 Sahlgrenska Cancer Center, University of Gothenburg, 405 30 Gothenburg, Sweden; 8 Breakthrough Breast Cancer Unit, School of Cancer and Imaging Sciences, Paterson Institute for Cancer Research, University of Manchester, Wilmslow Road, Manchester M20 4BX, UK

**Keywords:** Yes-associated protein, Breast cancer, Estrogen receptor, Luminal A, 11q deletion, Tamoxifen response, Independent prognostic factor

## Abstract

**Background:**

Yes-associated protein (YAP1) is frequently reported to function as an oncogene in many types of cancer, but in breast cancer results remain controversial. We set out to clarify the role of YAP1 in breast cancer by examining gene and protein expression in subgroups of patient material and by downregulating YAP1 *in vitro* and studying its role in response to the widely used anti-estrogen tamoxifen.

**Methods:**

YAP1 protein intensity was scored as absent, weak, intermediate or strong in two primary breast cancer cohorts (n = 144 and n = 564) and mRNA expression of YAP1 was evaluated in a gene expression dataset (n = 1107). Recurrence-free survival was analysed using the log-rank test and Cox multivariate analysis was used to test for independence. WST-1 assay was employed to measure cell viability and a luciferase ERE (estrogen responsive element) construct was used to study the effect of tamoxifen, following downregulation of YAP1 using siRNAs.

**Results:**

In the ER+ (Estrogen Receptor α positive) subgroup of the randomised cohort, YAP1 expression was inversely correlated to histological grade and proliferation (p = 0.001 and p = 0.016, respectively) whereas in the ER- (Estrogen Receptor α negative) subgroup YAP1 expression correlated positively to proliferation (p = 0.005). Notably, low YAP1 mRNA was independently associated with decreased recurrence-free survival in the gene expression dataset, specifically for the luminal A subgroup (p < 0.001) which includes low proliferating tumours of lower grade, usually associated with a good prognosis. This subgroup specificity led us to hypothesize that YAP1 may be important for response to endocrine therapies, such as tamoxifen, extensively used for luminal A breast cancers. In a tamoxifen randomised patient material, absent YAP1 protein expression was associated with impaired tamoxifen response which was significant upon interaction analysis (p = 0.042). YAP1 downregulation resulted in increased progesterone receptor (PgR) expression and a delayed and weaker tamoxifen in support of the clinical data.

**Conclusions:**

Decreased YAP1 expression is an independent prognostic factor for recurrence in the less aggressive luminal A breast cancer subgroup, likely due to the decreased tamoxifen sensitivity conferred by YAP1 downregulation.

## Background

The Yes-associated protein (YAP1) was discovered in 1994 as a binding partner of the SH3 domain of the Yes proto-oncogene product [1]. Since then, a vast number of publications describing the structure and function of this transcriptional co-regulator have been published (reviewed in [2]). The YAP1 protein contains several binding motifs which allow for protein-protein interactions; for example the WW domain (present in either single or dual form due to splicing events [3]) which can bind and regulate proteins by interaction with a proline rich PPxY motif. YAP1 also contains a TEAD binding domain necessary for activation of the TEAD transcription factors, which upon aberrant activation leads to increased cell growth and proliferation, ultimately resulting in tissue overgrowth [4-7]. In addition, the activation of TEAD by YAP1 is reported to result in oncogenic transformation of several cell types [8,9]. YAP1 has been reported to bind and modulate the transcriptional activities of several proteins such as Runx2, TEAD, p73, ErbB4, Smad7 and Smad1 [7,10-15].

To date, there are several reports on the function of YAP1 as an oncogene in breast cancer models, but tumour suppressive functions have also been reported. Overexpression of YAP1 leads to oncogenic transformation of the immortalised MCF10A human breast cell line [16] and the TEAD-interaction domain of a constitutively active YAP1^S127A^ mutant has been shown to promote tumour growth and metastasis of murine mammary carcinoma cell lines [17]. In addition, downregulation of YAP1 in the human breast cancer cell line MCF-7 resulted in decreased cell proliferation and complete loss of tumour formation in mice [18]. Similar results were obtained upon depletion of YAP1 in the basal-like SW527 human breast cancer cell line [19], altogether suggesting YAP1 to function as an oncogene in breast cancer. Furthermore, YAP1 is now widely recognized as one of the oncogenic drivers of 11q22 amplification in liver cancer [20,21] and in many other cancer forms such as ovarian, lung and esophageal squamous cell carcinoma, overexpression of YAP1 is correlated to a worse outcome [22-24].

Despite these reports pointing to YAP1 as an oncogene, the role of YAP1 in breast cancer is far from clear. Yuan and co-authors reported in 2008 that stable downregulation of YAP1 in breast cancer cell lines resulted in protection of anoikis, promotion of anchorage-independent growth and increased migration and invasion. YAP1 depletion resulted in increased tumour growth in nude mice, altogether suggesting a tumour suppressive function of YAP1 in breast cancer [25]. The chromosomal location of the *YAP1* gene at 11q22 is also in favour of it functioning as a tumour suppressor given the frequent loss of heterozygosity (LOH) and deletions of this region in breast cancers [26-30]. In addition, amplification of *YAP1* in human breast cancer is infrequent [16] and YAP1 protein expression is often decreased in primary breast cancer [25,31-33]. Therefore, it might be challenging to translate *in vitro* findings of YAP1 into a clinical setting. To our knowledge, there are no reports concerning the expression of YAP1 and correlations with outcome in subsets of breast cancer patients, hence we set out to investigate and clarify the role of YAP1 in breast cancer.

In this study, we have examined the expression of YAP1 both on protein and gene expression level in a total of 1751 primary breast cancer samples with clinical follow-up. We show that in ER+ breast cancer, decreased YAP1 expression is associated with more aggressive features such as higher histological grade, increased proliferation and lymph node positivity. In ER- breast cancer the relationship is opposite and increased YAP1 expression correlated to increased proliferation. Furthermore, low YAP1 mRNA expression is independently associated with a worse outcome in the luminal A molecular breast cancer subgroup. We suggest this result relates to a decrease in tamoxifen sensitivity which potentially results from the altered levels of estrogen receptor (ER) and progesterone receptor (PgR) observed upon YAP1 downregulation in the luminal breast cancer cell line T47D.

## Methods

### Patient data

Several patient cohorts were used in this study. The ‘screening cohort’ consisted of 144 women diagnosed with primary invasive breast cancer at Malmö University Hospital during the years of 2001 and 2002. Ethical permission was obtained from the Lund University Regional Ethics Board and written consent was not required. Median follow-up time for the patients was 5.75 years and median age at diagnosis was 65 years (range 35-97 years). All patients were treated following surgery. This cohort was originally designed as a first-line breast cancer screening cohort for Human Protein Atlas antibodies and further details of the material may be viewed in references [34,35].

The ‘randomised cohort’ consisted of 564 premenopausal patients presenting with invasive stage II breast cancer who were enrolled in a randomised controlled clinical trial, recruiting between the years of 1986 and 1991. The Lund University and Linköping University Regional Ethics Boards approved the initial randomised study, and there was no requirement for additional consent for the present study. Tumour material was available from 500 patients. The primary aim of the trial was to determine the effect of 2 years of tamoxifen treatment on recurrence-free survival compared to no treatment and patients were included regardless of ER status. Median follow-up time was 13.9 years and further details can be found in reference [36]. Out of the 500 available tumours from the randomised cohort, 324 were successfully evaluated for YAP1 expression. Analysis of the missing tumour cores showed a slight correlation to PgR positivity (Spearman’s rho 0.105, p = 0.024), a lower NHG grade (Spearman’s rho -0.110, p = 0.013) and a low Ki-67 expression (Spearman’s rho -0.122, p = 0.012). No differences were found in breast cancer recurrences comparing the two groups.

For the gene expression analysis of 1107 primary breast cancers, a meta-analysis of six comprised Affymetrix datasets was performed as previously described [37]. Endpoints for datasets Chin *et al.*, Pawitan *et al.* and Sotiriou *et al.* was recurrence-free survival and for Desmedt *et al.*, Ivshina *et al.* and Wang *et al.* datasets it was disease-free survival. In this study, we have referred to all endpoints as recurrence-free survival. The Affymetrix U133A probe set ID used for YAP1 was 213342_at. The classification of molecular breast cancer subgroups was made according to the Norway/Stanford signature [37]. Further details of the datasets included in the analysis can be found in references [38-43].

The aCGH (array Comparative Genomic Hybridisation) patient data set consisted of 171 patients with primary operable breast cancer. The dataset is publicly available from NCBI’s GEO under the series accession number GSE8757 and further details may be found in reference [44].

### Tissue microarray, immunohistochemical staining and scoring of YAP1 expression

Tumours from the screening and randomised cohorts were assembled in tissue microarrays using a manual tissue arrayer (MTA-1; BeecherInstruments, Inc., Sun Prairie, WI). The pre-treatment process of deparaffinization, rehydration and epitope retrieval of the 4 μm sections was carried out using the PT Link module (Dako, Glostrup, Denmark). Staining procedure with YAP1 antibody (1:25, Cell Signaling Technology Inc., Danvers, MA, cat#4912) was performed using the Autostainer Plus instrument with the Envision Flex programme (Dako). The epitope used for raising the YAP1 antibody includes amino acid 100 (personal communication, Cell Signaling Technology Europe B.V.) and should therefore detect all to date known isoforms of YAP1 [3]. YAP1 was scored as overall intensity as either absent, weak, intermediate or strong by a research associate (SL) and a pathologist (GL). Expression of ER, Ki-67, cyclin D1 and amplification of *CCND1* (randomised cohort) had been scored previously in both the randomised and screening cohorts [35,45,46].

### Cell culture and transfection

The human breast cancer cell line T47D (ATCC, Int., Manassas, VA) was maintained in DMEM high glucose medium supplemented with 10% fetal bovine serum (FBS), 1 mM sodium pyruvate, 2 mM L-glutamine and 1xPEST (streptomycin 90 μg/ml, penicillin 90 IU/ml). Twenty-four hours before transfection, cells were seeded in PEST-free media which was subsequently replaced by PEST-free serum-free media and siRNA solution (OptiMEM, Gibco, Lipofectamine 2000, Invitrogen Life Technologies, Carlsbad, CA), yielding a final siRNA concentration of 40 nM. For negative control, the ON-TARGETplus Non-targeting control siRNA #2 (#D-001810-02) was used and for targeting YAP1, two different siRNAs were used; ON-TARGETplus YAP1 #7 (#J-012200-07) and ON-TARGETplus YAP1 #8 (#J-012200-08) (Dharmacon, Thermo Fisher Scientific Inc., Waltham, MA). After five hours, transfection was discontinued by replacement of medium to regular serum medium.

### WST-1 cell viability assay

The effect of 17β-estradiol (E2) and 4-OH-tamoxifen was determined by use of WST-1 assay. T47D cells were seeded at a density of 400 000 cells in a 60 mm Ø culture dish (28.3 cm^2^) in PEST-free media and transfected the following day as described. Forty-eight hours after transfection, cells were re-seeded in phenol red-free DMEM supplemented with 5% charcoal stripped serum in a 96-well plate (5000 cells/well). After an additional 24 hours, cells were incubated at 37°C in phenol red-free DMEM supplemented with 1% charcoal stripped serum with either control treatment (EtOH), 1 nM 17β-estradiol (E2) (Sigma #E2758, Sigma-Aldrich Co, St. Louis, MO) or 1 nM E2 and increasing concentrations of 4-OH-tamoxifen (10 nM, 100 nM and 1 μM) (Sigma #H7904, Sigma-Aldrich Co), the active metabolite of tamoxifen, for 4 days. WST-1 assay reagent (Roche Applied Science, Mannheim, Germany) was subsequently added (10 μl) to each well and cells were incubated for 4 hours at 37°C before the absorbance of each well was measured at the wavelength of 450 nm and reference wavelength of 690 nm, using a scanning multiwell spectrophotometer (Synergy 2). Statistics were calculated using Student’s t-test assuming unequal variances and the mean ± SD (standard deviation) is presented. Each experiment was measured in triplicate and repeated five times.

### Western blotting and immunocytochemistry

For western blot analysis, cells were scraped in cold PBS and lysed in ice-cold lysis buffer (0.1% Triton X-100, 0.5% NaDOC, 0.1% SDS, 50 mM Tris-HCl pH 7, 150 mM NaCl, 1 mM EDTA, 1 mM NaF) supplemented with protease inhibitor cocktail Complete Mini and phosphatase inhibitor cocktail phosSTOP (Roche, Basel, Switzerland). Cell extracts were kept on ice for 30 minutes and vortexed every 10 min followed by centrifugation at 14 000 rpm for 30 min. Supernatants were subsequently collected and protein concentration was determined using the BSA Protein Assay kit (Pierce, Rockford, IL). Twenty μg of protein were separated on 10% SDS-PAGE gels and transferred onto nitrocellulose membranes (Hybond ECL, Amersham Pharmacia Biotech, Buckinghamshire, UK). Primary antibodies used included YAP1 (Cell Signaling Technology Inc., Danvers, MA, cat#4912), cyclin D1 (clone SP4, Dako, Glostrup, Denmark), cyclin A2 (H432, Santa Cruz Biotechnology Inc., Dallas, TX, cat#sc-751), and actin (I-19, Santa Cruz Biotechnology, Inc., Dallas, TX, cat#sc-1616). For immunocytochemistry, cells were trypsinised and fixed in 4% formaldehyde for 30 min followed by staining with Meyer’s haematoxylin for 5 min. Cells were subsequently centrifuged at 1400 rpm for 5 min and cell pellets were resuspended in 70% ethanol over night. Cell pellets were dehydrated in graded ethanol series, embedded in paraffin and a cell pellet array was constructed and stained using the following antibodies and dilutions: YAP1 (Cell Signaling Technology Inc., Danvers, MA, 1:25, cat#4912), ERα (clone 1D5, Dako, Glostrup, Denmark, 1:50, cat#M7047) and PgR (clone 636, Dako, 1:1500, cat#M3569). The experiment was repeated three times and one representative experiment was quantified by automated image analysis.

### Luciferase assay

T47D cells were seeded in a 12-well plate at a density of 100 000 cells per well and transfected with siCtr, siYAP1 #7 or siYAP1 #8 as described. Forty-eight hours after siRNA transfection, cells were re-transfected with 0.5 μg pGL2 luciferase reporter plasmid (pERE-luc) containing the ER binding element ERE (Estrogen Response Element) together with 0.2 μg of the Renilla expressing plasmid pRL-TK, which served as an internal control. Five hours later, transfection media was replaced by phenol red-free DMEM, supplemented with 5% charcoal stripped serum and PEST, and cells were kept in this media 24 hours prior to treatment initiation. Cells were subsequently treated with either 1 nM 17β-estradiol (E2) (Sigma #E2758, Sigma-Aldrich Co, St. Louis, MO) or 1 nM E2 and 100 nM 4-hydroxi-tamoxifen (4-OH-tam) combined (Sigma #H7904, Sigma-Aldrich Co). Ethanol was used as control treatment, mimicking the amount used for the E2 and E2 + 4-OH-tam wells. After 24 hours of treatment, luciferase activity was measured using the Dual-Luciferase® Reporter Assay System (Promega Corporation, Madison, WI) and normalised to the internal control. Three wells were included for each treatment in every experiment (n = 3) and luciferase measurements were made in triplicate.

### Statistics

To examine statistical associations of YAP1 and clinical and molecular parameters, the non-parametric Spearman’s rank correlation coefficient test and Mann-Whitney U test were employed. The p-values were not adjusted for multiple testing. Survival analysis was carried out using the Kaplan-Meier method and recurrence-free survival was compared by means of the log-rank test. The IBM SPSS software program (version 20.0, IBM Corporation, Armonk, NY) was used for calculation.

Statistical significance of differences in tamoxifen response in cell viability experiments (WST-1) and luciferase experiments were calculated using an unpaired two-tailed student’s t-test assuming equal variances, unless stated otherwise. Bars indicate the mean of at least three independent experiments and error bars designate ± SD. Results were considered significant if p < 0.05.

## Results

### YAP1 protein and mRNA expression in primary breast tumour materials and correlations to clinicopathological and molecular parameters

**Figure 1:**
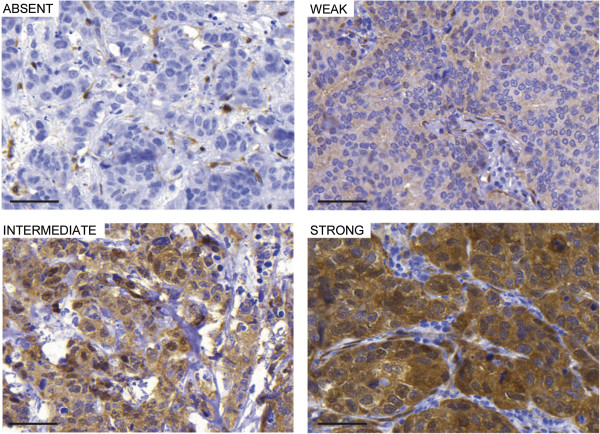
**YAP1 staining in primary breast cancers.** YAP1 overall intensity was scored as absent, weak, intermediate or strong. Scale bar = 50 μm.

YAP1 overall protein intensity was scored as either absent, weak, intermediate or strong (Figure 1) in two different primary breast cancer cohorts (screening cohort, n = 144 and randomised cohort, n = 500). YAP1 mRNA expression was also explored using a large gene expression dataset consisting of six previously published primary breast cancer datasets totalling 1107 patients [37]. There were no correlations regarding YAP1 expression and grade, lymph node status or tumour size when including both ER+ and ER- patients in the analysis of the two cohorts and the gene expression dataset (Tables 1, 2 and 3). We next divided our cohorts on the basis of estrogen receptor status. In the ER+ patient group of the screening cohort, an inverse correlation between YAP1 expression and lymph node involvement was observed (p = 0.022, Table 1) and in the ER+ subgroup of the randomised cohort, YAP1 expression was negatively correlated to proliferation (measured by Ki-67) and histological grade (p = 0.016 and p = 0.001 respectively) (Table 2). In contrast, in the ER- subgroup of the randomised cohort, a positive correlation between YAP1 expression and proliferation was observed illustrating the importance of performing subgroup analysis (p = 0.005) [see Additional file 1].

**Table 1:** Correlations of YAP1 protein expression and clinical and molecular parameters of the screening cohort (n=144)

	**All patients, n=144**					**ER**^**+ **^**patients, n=125**				
	**YAP1 intensity, n=117**					**YAP1 intensity, n=99**				
	**Absent**	**Weak**	**Intermediate**	**Strong**		**Absent**	**Weak**	**Intermediate**	**Strong**	
	**n (%)**	**n (%)**	**n (%)**	**n (%)**	**p-value**	**n (%)**	**n (%)**	**n (%)**	**n (%)**	**p-value**
**Variable**	**4 (3)**	**52 (44)**	**38 (33)**	**23 (20)**		**4 (4)**	**46 (47)**	**35 (35)**	**14 (14)**	
NHG										
I	0	3 (6)	6 (16)	2 (9)		0	3 (7)	6 (17)	2 (14)	
II	2 (50)	26 (50)	19 (50)	9 (41)		2 (50)	25 (54)	19 (54)	9 (64)	
III	2 (50)	23 (44)	13 (34)	11 (50)	0.646^a^	2 (50)	18 (39)	10 (29)	3 (21)	0.060^a^
Lymph node status										
Negative	1 (25)	26 (52)	22 (65)	12 (63)		1 (25)	23 (51)	20 (63)	10 (83)	
Positive	3 (75)	24 (48)	12 (35)	7 (37)	0.128^b^	3 (75)	22 (49)	12 (37)	2 (17)	0.022^b^
Tumour size										
<20 mm	0	26 (50)	18 (47)	9 (41)		0	24 (55)	18 (51)	8 (57)	
≥20 mm	4 (100)	26 (50)	20 (53)	13 (59)	0.995^b^	4 (100)	22 (45)	17 (49)	6 (43)	0.347^b^
ERα										
<10%	0	6 (12)	3 (8)	8 (36)		-	-	-	-	
≥10%	4 (100)	46 (88)	35 (92)	14 (64)	0.030^b^	-	-	-	-	
PgR										
<10%	1 (25)	17 (33)	9 (24)	14 (64)		1 (25)	12 (26)	6 (17)	6 (43)	
≥10%	3 (75)	35 (67)	29 (76)	8 (36)	0.084^b^	3 (75)	34 (74)	29 (83)	8 (57)	0.691^b^
Ki-67 fraction (%)										
0-10	0	0	3 (8)	2 (10)		0	0	3 (9)	2 (15)	
11-25	2 (67)	20 (44)	18 (49)	7 (33)		2 (67)	18 (45)	18 (53)	6 (46)	
26-100	1 (33)	25 (56)	16 (43)	12 (57)	0.626^a^	1 (33)	22 (55)	13 (38)	5 (39)	0.091^a^
Cyclin D1 intensity										
Negative/Low	0	19 (51)	20 (67)	12 (86)		0	17 (53)	17 (63)	6 (75)	
Moderate/High	4 (100)	18 (49)	10 (33)	2 (14)	0.002^b^	4 (100)	15 (47)	10 (37)	2 (25)	0.040^b^

NHG=Nottingham histological grade, ER=estrogen receptor, PgR=progesterone receptor.

^a^Spearman´s rank correlation.

^b^Mann-Whitney U test.

**Table 2:** Correlations of YAP1 protein expression and clinical and molecular parameters of the randomised cohort (n=500)

	**All patients, n= 500**					**ER**^**+ **^**patients, n=324**				
	**YAP1 intensity, n=324**					**YAP1 intensity, n=213**				
	**Absent**	**Weak**	**Intermediate**	**Strong**	**p-value**	**Absent**	**Weak**	**Intermediate**	**Strong**	**p-value**
	**n (%)**	**n (%)**	**n (%)**	**n (%)**		**n (%)**	**n (%)**	**n (%)**	**n (%)**	
**Variable**	**28 (9)**	**130 (40)**	**128 (39)**	**38 (12)**		**21 (10)**	**95 (45)**	**79 (37)**	**18 (8)**	
NHG										
I	0	10 (8)	11 (9)	5 (13)		0	10 (11)	11 (14)	5 (28)	
II	13 (48)	55 (44)	56 (45)	10 (27)		12 (57)	48 (50)	51 (65)	9 (50)	
III	14 (52)	60 (48)	57 (46)	22 (60)	0.932^a^	9 (43)	37 (39)	16 (21)	4 (22)	0.001^a^
Lymph node status										
Negative	7 (25)	35 (27)	34 (27)	15 (41)		5 (24)	25 (26)	19 (24)	5 (29)	
Positive	21 (75)	94 (73)	94 (73)	22 (59)	0.285^b^	16 (76)	70 (74)	60 (76)	12 (71)	0.971^b^
Tumour size										
<20 mm	12 (44)	43 (33)	46 (36)	13 (34)		10 (50)	35 (37)	33 (42)	6 (33)	
≥20 mm	15 (56)	87 (67)	82 (64)	25 (66)	0.815^b^	10 (50)	60 (63)	46 (58)	12 (67)	0.757^b^
ERα										
<10%	4 (16)	33 (26)	46 (37)	20 (53)		-	-	-	-	
≥10%	21 (84)	95 (74)	79 (63)	18 (47)	<0.001^b^	-	-	-	-	
PgR										
<10%	6 (24)	35 (29)	47 (39)	20 (56)		1 (5)	6 (7)	4 (5)	0	
≥10%	19 (76)	86 (71)	75 (61)	16 (44)	0.002^b^	18 (95)	84 (93)	73 (95)	16 (100)	0.464^b^
Ki-67 fraction (%)										
0-10	12 (48)	48 (40)	53 (45)	10 (29)		9 (47)	37 (42)	47 (64)	9 (56)	
11-25	7 (28)	38 (32)	34 (29)	7 (20)		6 (32)	30 (34)	20 (27)	3 (19)	
26-100	6 (24)	34 (28)	31 (26)	18 (51)	0.198^a^	4 (21)	21 (24)	6 (8)	4 (25)	0.016^a^
Cyclin D1 intensity										
absent/weak	8 (31)	67 (53)	81 (67)	29 (76)		3 (14)	39 (42)	40 (51)	9 (50)	
intermediate/strong	18 (69)	59 (47)	40 (33)	9 (24)	<0.001^b^	18 (86)	54 (58)	38 (49)	9 (50)	0.010^b^
*CCND1* amplification										
No	8 (44)	69 (84)	75 (89)	24 (96)		5 (36)	48 (80)	47 (84)	12 (92)	
Yes	10 (56)	13 (16)	9 (11)	1 (4)	<0.001^b^	9 (64)	12 (20)	9 (16)	1 (8)	0.003^b^

NHG=Nottingham histological grade, ERα=estrogen receptor, PgR=progesterone receptor.

^a^Spearman´s rank correlation.

^b^Mann-Whitney U test.

**Table 3:** Correlations of YAP1 mRNA expression and clinical and molecular parameters of the gene expression dataset (n=1107)

	**All patients, n=1107**					**ER+ patients, n=700**				
	**YAP1 mRNA Quartiles**					**YAP1 mRNA Quartiles**				
	**Q1**	**Q2**	**Q3**	**Q4**		**Q1**	**Q2**	**Q3**	**Q4**	
**Variable**	**n=277 (%)**	**n=276 (%)**	**n=277 (%)**	**n=277 (%)**	**p-value**	**n=175 (%)**	**n=175 (%)**	**n=175 (%)**	**n=175 (%)**	**p-value**
NHG										
I	29 (15)	43 (24)	46 (24)	49 (24)		17 (15)	34 (31)	33 (28)	38 (30)	
II	94 (47)	79 (43)	77 (40)	80 (38)		60 (51)	48 (44)	54 (46)	48 (37)	
III	77 (38)	61 (33)	69 (36)	80 (38)	0.355^a^	40 (34)	27 (25)	31 (26)	42 (33)	0.165^a^
Lymph node status										
Negative	187 (81)	207 (87)	194 (80)	192 (85)		138 (80)	155 (89)	139 (81)	138 (79)	
Positive	44 (19)	31 (13)	47 (20)	35 (15)	0.715^b^	34 (20)	20 (11)	32 (19)	36 (21)	0.433^b^
Tumour size										
<20 mm	78 (48)	85 (54)	93 (54)	82 (48)		55 (45)	59 (52)	72 (59)	76 (56)	
≥20 mm	86 (52)	72 (46)	78 (46)	88 (52)	0.917^b^	66 (55)	54 (48)	50 (41)	59 (44)	0.056^b^
ERα										
<10%	44 (19)	57 (24)	56 (23)	82 (36)		-	-	-	-	
≥10%	188 (81)	180 (76)	187 (77)	145 (64)	<0.001^b^	-	-	-	-	
PgR Quartiles										
Q1	66 (24)	62 (22)	60 (22)	89 (32)		51 (29)	45 (26)	36 (21)	43 (25)	
Q2	82 (30)	76 (28)	60 (22)	59 (21)		49 (28)	48 (27)	41 (23)	37 (21)	
Q3	76 (27)	70 (25)	71 (25)	60 (22)		40 (23)	43 (25)	48 (27)	44 (25)	
Q4	53 (19)	68 (25)	86 (31)	69 (25)	0.836^a^	35 (20)	39 (22)	50 (29)	51 (29)	0.011^a^
Cyclin D1 Quartiles										
Q1	57 (20)	73 (26)	66 (24)	81 (29)		31 (18)	45 (26)	51 (29)	48 (27)	
Q2	41 (15)	63 (23)	87 (31)	85 (31)		26 (15)	47 (27)	53 (30)	49 (28)	
Q3	69 (25)	69 (25)	78 (28)	61 (22)		47 (27)	33 (19)	44 (25)	51 (29)	
Q4	110 (40)	71 (26)	46 (17)	50 (18)	<0.001^a^	71 (41)	50 (28)	27 (16)	27 (16)	<0.001^a^

NHG=Nottingham Histological Grade, ER=Estrogen receptor, PgR=Progesterone receptor.

^a^Spearman´s rank correlation.

^b^Mann–Whitney U test.

Furthermore, YAP1 expression was inversely linked to ER and cyclin D1 expression in all three patient cohorts. When dividing the materials according to ER status, the inverse correlation between YAP1 and cyclin D1 only remained in the ER+ subgroups (Tables 1, 2 and 3, Additional files 1 and 2). In the gene expression dataset, YAP1 mRNA quartiles were positively correlated to tumour size in the ER- subgroup (p = 0.037) [see Additional file 2].

Taken together, in ER+ tumours low YAP1 expression is linked to more clinically aggressive features including grade and proliferation. In ER- tumours the relationship is reversed and high YAP1 expression was linked to more aggressive features.

### YAP1 loss and *CCND1* amplification are inversely correlated in patient materials

The *YAP1* gene is located at 11q22, a region often deleted upon amplification of the 11q13 region harbouring the known oncogene cyclin D1 gene (*CCND1*), which is amplified in 8-15% of all breast cancers and associated with a worse prognosis [47-49]. The inverse correlation seen between YAP1 and cyclin D1 protein and mRNA expression could be due to a recurring chromosomal rearrangement, resulting in overexpressed cyclin D1 (following amplification) and decreased YAP1 protein expression (following deletion). *CCND1* amplification had previously been assessed in the randomised cohort (for further details, see ref [46]) and 9/14 ER+ patients (64%) with absent YAP1 expression also had amplification of *CCND1* (Table 2). However, when removing the *CCND1* amplified cases from the analysis, the inverse correlation between YAP1 and cyclin D1 protein expression in the ER+ subgroup remained (Spearman’s rho -0.206, p = 0.030) indicating additional mechanisms for maintaining the negative relationship. This was despite the fact that *CCND1* amplified cases were associated with a stronger cyclin D1 expression in this material (data not shown).

The inverse correlation of *CCND1* and *YAP1* was further examined in an aCGH dataset. Amplification of *CCND1* was frequently associated with loss of *YAP1* [see Additional file 3]*.* Nonetheless, amplification of *CCND1* was not a prerequisite for *YAP1* gene loss, as there were several tumours with low *YAP1* copy number where increased *CCND1* copy numbers were not present [Additional file 3b, lower panel].

To summarise, *CCND1* amplification is associated with *YAP1* gene loss but the negative association between the proteins is not entirely dependent on chromosomal rearrangements, as the correlation remains after removing cases of *CCND1* amplification. Furthermore, *YAP1* gene loss may occur independently of *CCND1* amplification.

### YAP1 mRNA expression holds independent prognostic value

**Figure 2:**
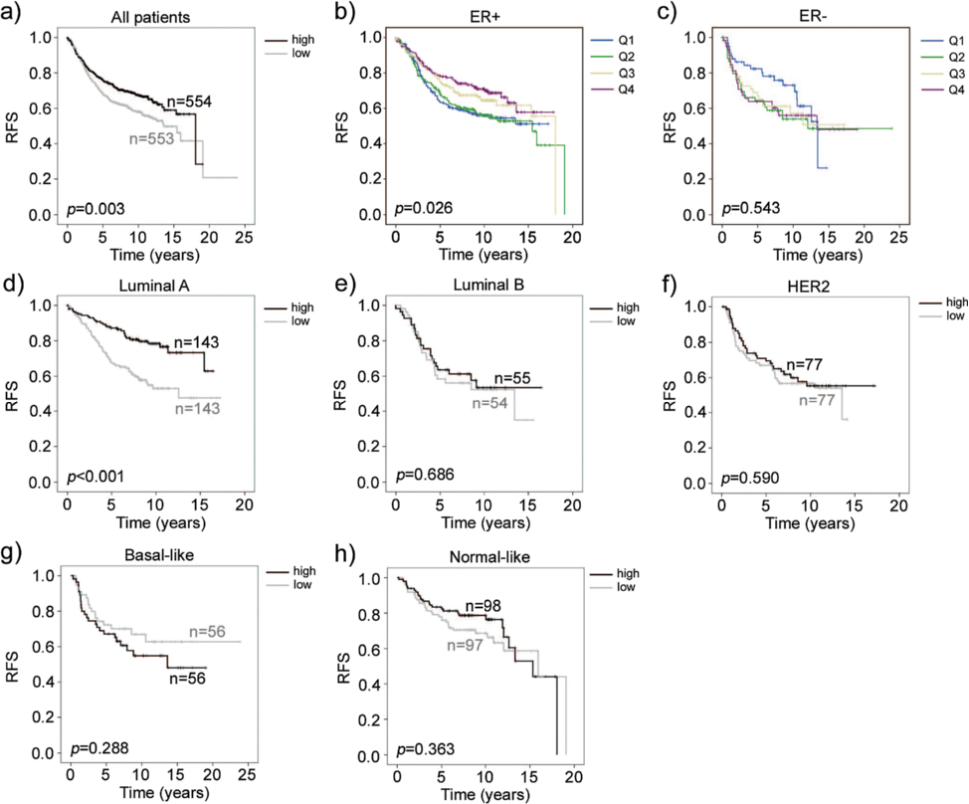
**YAP1 mRNA predicts outcome in molecular subgroups of primary breast cancer. (a)** YAP1 mRNA expression was dichotomised at the median value to generate high and low YAP1 expressing groups. Low YAP1 mRNA correlates to a decreased recurrence-free survival in the entire dataset. **(b)** YAP1 mRNA quartiles of the ER+ subgroup (n = 700) correlates to a decreased recurrence-free survival whereas the trend in the **(c)** ER- subgroup (n = 239) is opposite but not significant. **(d-h)** Survival analyses in breast cancer molecular subgroups. Low YAP1 mRNA is associated with decreased recurrence-free survival in the luminal A but not luminal B, HER2, basal or normal-like subgroups. Q = quartile, RFS = recurrence-free survival.

In order to investigate the influence of YAP1 expression on disease outcome, survival analyses were performed. In the screening cohort, YAP1 expression was not associated with recurrence-free survival [see Additional file 4]. The gene expression dataset was analysed for recurrence using the median of YAP1 mRNA expression as a cut-off to define groups of high or low YAP1 expression (Figure 2a). Low YAP1 mRNA expression was correlated to a decreased recurrence-free survival and YAP1 mRNA proved to be an independent prognostic factor after adjustment for known prognostic factors such as grade, tumour size and lymph node involvement [see Additional file 5].

As correlations in the screening and randomised patient cohorts implied that YAP1 behaves differently depending on the tumours’ expression of ER, recurrence-free survival was analysed in ER+ and ER- subgroups of the gene expression dataset (Figure 2b and c). In the ER+ subgroup, the two lower quartiles correlated to a shorter recurrence-free survival. Interestingly, in the ER- subgroup the trend was the opposite. Quartiles 2, 3 and 4 were in the bottom of the graph whereas quartile 1 (holding the tumours of lowest YAP1 mRNA expression) demonstrated a remarkably better outcome after 5 years of follow-up, although the trend was not persistent. These results are well in line with the correlations in Tables 1, 2 and 3, implying a contrasting function of YAP1 in breast cancer subgroups.

### Low YAP1 mRNA expression is specifically correlated to worse outcome in the luminal A breast cancer subgroup

We further explored if YAP1 mRNA had different significance in regards to outcome in breast cancer molecular subgroups. Strikingly, low YAP1 mRNA was only of importance in the luminal A subtype and of no importance in the remaining four subtypes (luminal B, HER2, basal-like and normal-like) when dividing the dataset accordingly (Figure 2d-h). Subgroup analysis of YAP1 mRNA expression showed that expression was significantly higher in the normal-like and basal-like subgroups compared to the luminal A and B subgroups. However, no statistical difference was found between luminal A and B subgroups [see Additional file 6].

Due to the frequent deletion of the 11q22 chromosomal region, several genes in close proximity to *YAP1* were tested for correlation and outcome in the gene expression dataset [see Additional file 7]. However, YAP1 was the only factor which remained significant of outcome in the multivariate analysis for the luminal A subgroup [see Additional file 8].

In conclusion, decreased YAP1 mRNA expression is a prognostic factor in the luminal A subgroup, independent of a selection of proximal 11q22 genes, cyclin D1 or established prognostic factors.

### Absence of YAP1 protein expression in primary breast tumours is linked to an impaired tamoxifen response

**Figure 3:**
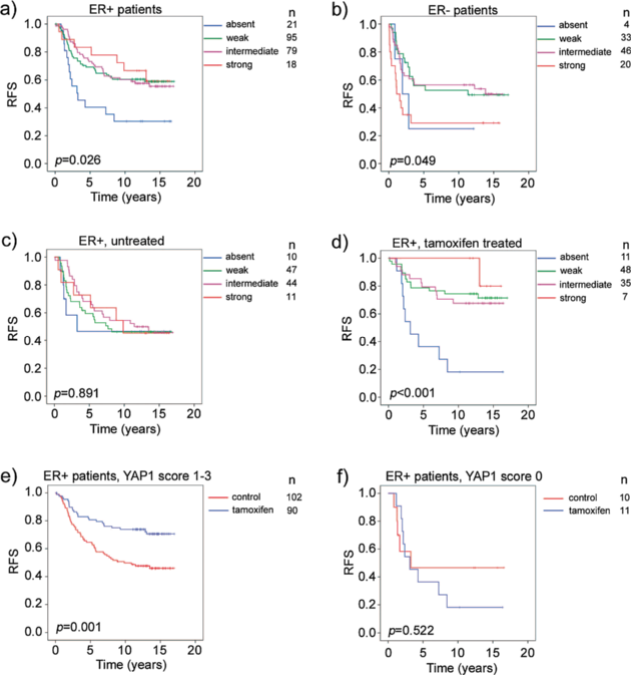
**Absence of YAP1 protein expression is associated with tamoxifen resistance in the randomised patient cohort. (a)** Kaplan-Meier analysis of all ER+ patients, both treated (tamoxifen) and untreated. Absent YAP1 expression is correlated to a decreased recurrence-free survival. **(b)** In the ER- subgroup both absent and strong YAP1 expression correlated to a worse outcome. **(c)** Analysis of the untreated patient cohort of ER+ patients indicates no prognostic value of YAP1. **(d)** Analysis of the tamoxifen treated patient cohort of ER+ patients suggests predictive value of absent YAP1. **(e)** ER+ patients with tumours scored as weak, intermediate or strong YAP1 expression do significantly better when treated with tamoxifen compared to the untreated control group whereas **(f)** ER+ patients with YAP1 scored as absent do not benefit from tamoxifen. RFS = recurrence-free survival.

The prominent effect of decreased YAP1 mRNA in the luminal A subtype led us to hypothesize that YAP1 could be important for the response to endocrine therapies. The majority of luminal A classified tumours are ER+ and hence treated with some variant of endocrine targeting treatment such as tamoxifen. To study the possible effect of YAP1 loss on tamoxifen response, recurrence-free survival was analysed in the randomised cohort, as this patient material originates from a clinical trial evaluating tamoxifen response in a randomised setting. Significance of YAP1 expression was initially analysed in ER+ and ER- subgroups, as molecular subgroup data was not available for this cohort. Figure 3a, which included both treated and untreated ER+ patients, showed significantly decreased recurrence-free survival when YAP1 expression was absent. In the ER- subgroup, both absent and strong YAP1 expression correlated to a worse outcome (Figure 3b). ER+ patients were then divided according to whether they received tamoxifen or control treatment (Figure 3c and d). YAP1 expression was not correlated to outcome in the untreated ER+ patient subgroup whereas there were significant differences in outcome in the tamoxifen treated ER+ subgroup. Figure 3e and f specifically address the tamoxifen response. ER+ patients with tumours of YAP1 expression scored as either weak, intermediate or strong (score 1-3) did significantly better when treated with tamoxifen compared to no treatment. In the group of patients with tumours of absent YAP1 expression (score 0), there was no difference in outcome between the control and tamoxifen group. A multivariate interaction analysis further demonstrated a statistically significant association between absent YAP1 and an impaired response to tamoxifen (p = 0.042, Table 4). These results suggest YAP1 as a predictive marker for tamoxifen response.

**Table 4:** The difference in treatment response between patient groups of different YAP1 expression is significant: A multivariate Cox proportional hazards regression analysis for YAP expression and treatment interaction based on ER+ breast cancer patients*

		**Recurrence-free survival**		
**Variable**	**Category**	**HR**	**95% CI**	***P *** **value**
YAP1 expression	Weak, intermediate or strong	1.00		
	Absent	1.42	0.56 to 3.59	0.456
Treatment	Control	1.00		
	Tamoxifen	0.42	0.26 to 0.68	<0.001
Interaction variable^†^	Tamoxifen x YAP1 expression	3.51	1.05 to 11.75	0.042

*Other factors included in the analysis are tumour grade (NHG I + II vs. III), nodal status (negative vs. positive) and tumor size (≤ 20 mm vs. >20 mm).

^†^Interaction variable states if there is a difference in the treatment response in relation to YAP1 expression.

HR = Hazard ratio, CI = Confidence interval, NHG = Nottingham histological grade.

### YAP1 downregulation in the luminal cell line T47D results in a weaker tamoxifen response

**Figure 4:**
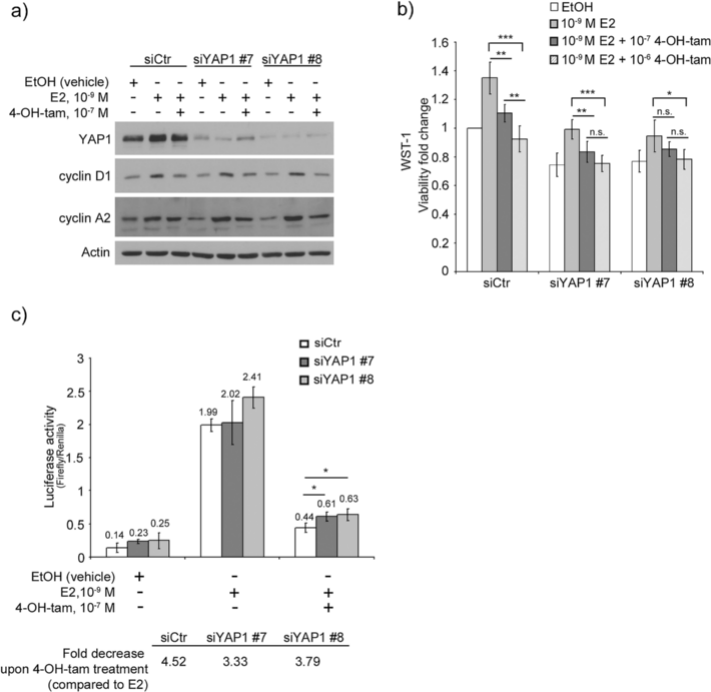
**Downregulation of YAP1 results in a weaker tamoxifen response in the T47D cell line.** T47D cells were transfected with siCtr, siYAP1 #7 or siYAP1 #8 and 48 h later medium was changed to phenol red-free DMEM supplemented with 5% charcoal stripped serum. Twenty-four hours later, the indicated treatment was added and after 4 days, cells were analysed for **(a)** YAP1, cyclin D1 and cyclin A2 protein expression by western blot **(b)** viability by WST-1 assay. Treatment of T47D results in a significant tamoxifen response for siCtr and siYAP1 #7 at 10^-7^ M 4-OH-tam whereas siYAP1 #8 was not significantly affected until 10^-6^ M 4-OH-tam. SiCtr cells respond significantly better to increasing concentrations of 4-OH-tam (**p = 0.006). No significant changes in response was observed when increasing 4-OH-tam in siYAP1 downregulated cells (siYAP1 #7, p = 0.09 and siYAP1 #8, p = 0.10). All values were related to EtOH treated siCtr cells. **(c)** T47D cells were transfected with siCtr, siYAP1 #7 or siYAP1 #8 followed by transfection with pERE-luc and pRL-TK as internal control. The mean value ± SD is indicated and fold decrease upon tamoxifen treatment (compared to E2 activation) is shown below the graph. P-values were calculated using paired student’s t-test (two-sided). *p < 0.05, **p < 0.01, ***p < 0.001. 4-OH-tam = 4-hydroxi-tamoxifen, E2 = 17β-estradiol, n.s. = no significance.

The T47D cell line was chosen to further investigate the role of YAP1 in tamoxifen response due to its relatively high expression of YAP1 compared to other luminal cell lines such as MCF-7, and also since proliferation in this cell line is not significantly affected by YAP1 downregulation [see Additional file 9] [18,50]. YAP1 was transiently downregulated followed by treatment with 17β-estradiol (E2) or E2 and increasing concentrations of 4-OH-tamoxifen. Cell viability was subsequently evaluated by means of WST-1 assay. YAP1 protein levels were efficiently downregulated and maintained at a depleted level even after 4 days of treatment (Figure 4a). There were no notable differences in the expression of the cell cycle proteins cyclin D1 and cyclin A2 when YAP1 was downregulated, although a slight decrease in cyclin A2 was noted in the EtOH control treated cells following YAP1 silencing (Figure 4a), compared to siCtr cells. To evaluate tamoxifen response, cell viability fold change was calculated comparing different concentrations of tamoxifen to estrogen stimulation, within a cell population treated with a specific siRNA (Figure 4b). Both siCtr and siYAP1 #7 demonstrated significant changes in cell viability upon addition of 10^-7^ M 4-OH-tamoxifen, whereas for siYAP1 #8, 4-OH-tamoxifen had no significant effect until the concentration of 10^-6^ M was reached. SiCtr treated cells responded significantly better to rising concentrations of 4-OH-tamoxifen (p = 0.006) whereas siYAP #7 and #8 showed no such dependence (p = 0.09 and p = 0.10, respectively).

To more specifically address the activity of ER, a luciferase assay measuring the activation of the estrogen response element (ERE) was employed. T47D cells were first transfected with siCtr, siYAP1 #7 or siYAP1 #8 followed by a pERE-luciferase construct transfection, and treated with E2 or a combination of E2 and 4-OH-tamoxifen for 24 hours (Figure 4c). Downregulation of YAP1 resulted in a less efficient tamoxifen-induced inhibition of ER activity, where siCtr cells showed a 4.52 fold decrease compared to only 3.33 and 3.79 for siYAP1 #7 and #8 cells, respectively.

To summarise, although a response to tamoxifen was still measurable, downregulation of YAP1 in the T47D cell line resulted in a later and less efficient tamoxifen response.

### Downregulation of YAP1 results in increased ER and PgR protein levels

**Figure 5:**
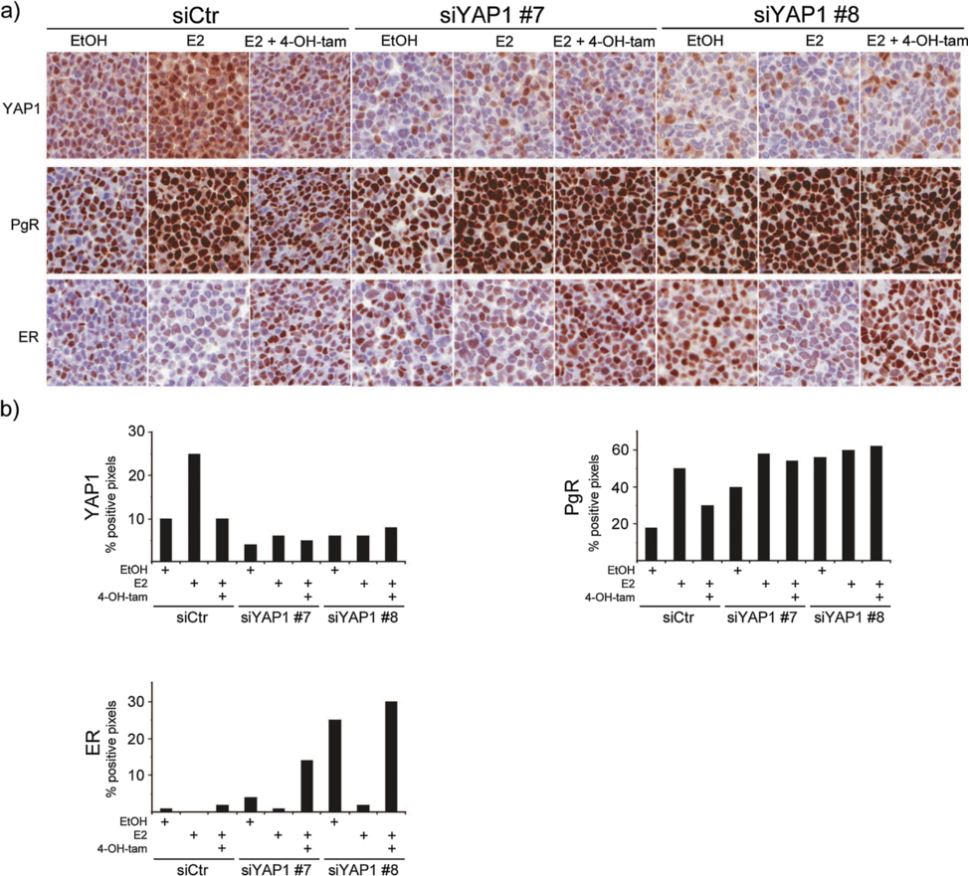
**ER and PgR protein levels increase upon YAP1 downregulation. (a)** Immunocytochemical stainings of the T47D cell line. Cells were transfected with the indicated siRNA and 48 hours later, medium was changed to phenol red-free DMEM supplemented with 5% charcoal stripped serum in which cells were grown for 24 hours. Subsequently, 10^-9^ M E2 or 10^-9^ M E2 combined with 10^-7^ M 4-OH-tam was added and 4 days later cells were harvested, fixed and stained using the indicated antibody. **(b)** Quantification by automated image analysis of stained cell pellets. Bars represent the percentage of positive pixels identified in the image analysis. ER = estrogen receptor α, PgR = progesterone receptor, 4-OH-tam = 4-hydroxi-tamoxifen, E2 = 17β-estradiol.

As ER and PgR protein expression are of great importance in predicting response to tamoxifen [51], T47D cell pellets (siCtr, siYAP1 #7 and #8) treated with EtOH, E2 or E2 and 4-OH-tamoxifen combined were examined for ER and PgR protein expression by immunocytochemistry (Figure 5). The knockdown of YAP1 was not 100% complete but the increase of YAP1 protein expression seen in siCtr cells upon E2 stimulation was effectively inhibited in siYAP#7 and #8 cells. Interestingly, siYAP1 #7 and #8 displayed a strong overall increase in PgR intensity, even in control treated (EtOH) cells. The decrease of PgR in 4-OH-tamoxifen treated siCtr cells was not as evident in siYAP1 #7 and #8 cells. As previously reported, ER is downregulated upon E2 treatment, an effect antagonised by tamoxifen which stabilises ER [52]. Although this effect was seen in all siRNA treatments, the expression of ER was higher overall in siYAP #7 and #8. Taken together, downregulation of YAP1 increased the level of hormone receptors, indicating deregulation of hormone receptor signalling when YAP1 is decreased.

## Discussion

In this study, we have shown that decreased mRNA levels of YAP1 independently predict outcome in ER+ and more specifically, luminal A breast cancers. This subgroup specificity led us to hypothesize that YAP1 is of importance in the response to endocrine treatments used to target the estrogen receptor, such as the widely used anti-estrogen tamoxifen. By examining a premenopausal primary breast tumour material randomised to either tamoxifen or control treatment, we found that absent YAP1 protein expression was associated with impaired tamoxifen response. Although effects were small, downregulation of YAP1 in a luminal breast cancer cell line resulted in a weaker tamoxifen response as measured by cell viability and activity of the estrogen receptor. In addition, silencing of YAP1 resulted in increased protein levels of ER and PgR, indicating increased signalling and deregulation of the ER pathway, a possible mechanism for the weaker tamoxifen response in this cell line.

The role of YAP1 in breast cancer is at present a matter of debate. By placing the protein in the context of estrogen receptor positive or negative disease, some reported results which appear contradictory may be explained. In 2006, Overholtzer *et al*. published a report on the oncogenic properties of YAP1, which had been identified through a screen for copy number changes in mouse mammary tumours. Interestingly, the genetic background used for screening was the *Brca1/Trp-53* which in the majority of cases gives rise to ER- mammary tumours of high grade [53]. The normal cell line subsequently used for transformation experiments was MCF-10A, reported to be ER- [54], altogether suggesting a possible function of YAP1 as an oncogene in ER- breast cancer. This is in support of our results where strong YAP1 expression was associated with increased proliferation in ER- tumours [see Additional file 1]. Furthermore, YAP1 was recently reported to function as an oncogene by promoting proliferation and survival of breast cancer cells by binding and stabilising the KLF5 (Kruppel-like factor 5) transcription factor through its PPxY motif [19]. KLF5 and YAP1 were reported to be predominately expressed in ER- cell lines and all experimental data was obtained from ER- breast cancer cell lines. Activation of YAP1 in the ER- cell line SUM159 [55] mediated by LIFR (leukemia inhibitory factor receptor) repression resulted in substantial lung metastases in nude mice, again suggesting an oncogenic role of YAP1 in ER- breast cancer [56]. The fundamental differences on the transcriptomic level of ER+ and ER- breast cancers is long established [57], and our results indicate that subgroup analysis is critical for the translational understanding of YAP1 in breast disease. There are however also reports where YAP1 show oncogenic features in ER+ breast cancer models. For example, stable knockdown of YAP1 in the ER+ human breast cancer cell line MCF-7 resulted in complete loss of tumour formation in BALB/c nude mice [18]. As YAP1 knockdown significantly decreased proliferation in MCF-7 cells, the negative result in tumour formation was perhaps expected. In the randomised breast cancer material studied here, decreased YAP1 expression was associated with increased proliferation in ER+ breast cancers (Table 2), indicating a discrepancy between the ER+ cell line model and primary breast cancer data. The effects of protein downregulation in cell line models are usually assessed after days or weeks, whereas primary tumours evolve during a period of years. As YAP1 loss is proposed to be an early event in breast cancer [25], YAP1 downregulation in cell lines such as MCF-7 might have to be assessed at much later time points in order to better correlate findings between primary tumour data and cell lines. The microenvironment might also constitute a critical parameter for understanding and modelling YAP1 in breast cancer, and stable downregulation of YAP1 in MCF-7 has been reported to result in increased invasion in a matrigel transwell assay [25].

The notion of YAP1 functioning as a tumour suppressor in breast cancer was first proposed by Yuan *et al*. in 2008 [25]. The 11q22 region where the *YAP1* gene is located is a frequent site of LOH (loss of heterozygosity) in breast cancer [26-30] and Yuan *et al.* reported that half of tumours negative for YAP1 staining also had specific LOH at the *YAP1* gene locus. This indicates 11q22 deletions as part of the explanation to the recurrent decreased YAP1 protein levels in breast cancer, but also supports the possibility of additional unknown mechanisms contributing to YAP1 protein loss in breast cancer [25,31-33]. Furthermore, downregulation of YAP1 was shown to increase anoikis, migration and invasion, altogether suggesting a tumour suppressive function for YAP1 [25]. In our study, YAP1 expression was not informative of outcome in the untreated ER+ subgroup of the randomised cohort (Figure 3c) which might be expected if YAP1 had had true tumour suppressive properties. In the ER+ tamoxifen treated subgroup, there were great differences in outcome depending on YAP1 expression (Figure 3d). Altogether, these results suggest YAP1 to function as a treatment predictive factor in ER+ breast cancer, rather than a prognostic factor for the natural disease progression.

*CCND1* amplification has previously been linked to a poor tamoxifen response, however cyclin D1 protein expression could not predict response as efficiently as the amplification event [46]. This indicates additional events affecting tamoxifen response associated with *CCND1* amplification. Co-amplification of other 11q13 genes is most likely of importance, but deletions of distal 11q might also contribute to tamoxifen resistance [58] and YAP1 is a likely candidate considering the data presented herein. *CCND1* amplified breast tumours have been reported to display loss of the distal part of 11q in 70% of cases [59]. However, the inverse correlation of cyclin D1 and YAP1 protein expression observed in the randomised tumour material remained after removing the *CCND1* amplified cases from the analysis, indicating additional functions for maintaining the negative relationship between the two proteins. Also, it has been reported that loss of distal 11q may occur independently of 11q13 amplification [26] as shown in [Additional file 3b] where *YAP1* loss is present independently of *CCND1* amplification in several cases (red arrows). Hence, our results suggest that loss of YAP1 may confer aggressiveness in ER+ breast cancers independently of the established oncogenic *CCND1* amplification.

The differences in correlations between the two patient cohorts might partly be explained by dissimilarities of the cohorts. The randomised patient cohort only included premenopausal patients with stage II breast cancer which renders this a more defined group compared to the screening cohort, where all patients with breast tumours defined as invasive was included. In the much smaller screening cohort, only four patients were assessed to have absent YAP1 protein expression, challenging the analysis since the complete loss of YAP1 appeared to be the conclusive factor on protein level. The conformity in treatment of the randomised cohort was further valuable for investigating the effect of YAP1 on tamoxifen response, whereas patients in the screening cohort were treated according to guidelines.

YAP1 has previously been reported to be able to enhance the hormone dependent activation of ER and PgR by acting as a coactivator through direct interaction with WW domain binding protein-2, WBP-2 [60]. The coactivation function of YAP1 was shown to be strictly dependent on the presence of WBP-2 which binds the estrogen receptor in a complex with E6-AP (E6-associated protein) upon E2 stimulation. From this perspective, downregulation of YAP1 would potentially lead to a decreased activation of ER in our experimental setting. Instead, the trend is an increase in ER signalling upon YAP1 downregulation illustrated by the increase in PgR protein levels in Figure 5, given that PgR is a known target gene of E2 signalling [61]. Interestingly, stabilisation of PgR has previously been shown to be important in *Brca1*-mediated tumorigenesis [62,63]. Also, no effect was observed on the E2 dependent activation of the Estrogen Responsive Element (ERE) upon depletion of YAP1. If any effect, the activity of the ERE is increased (siYAP1 #8, Figure 4c).

*In vitro* experiments downregulating YAP1 in the luminal A classified T47D cell line resulted in a weaker response to tamoxifen as measured by cell viability. There were several reasons for choosing the T47D cell line for the modelling of tamoxifen response. First, T47D cells have a higher gene and protein expression of YAP1 compared to other luminal breast cancer cell lines such as MCF-7, making the T47D cell line more suitable for YAP1 downregulating experiments [see Additional file 9c] [18,50]. Second, T47D cells are not as reliant on YAP1 for proliferation as MCF-7 cells [see Additional file 9a and b] which also favours the selection of the T47D cell line. In Figure 4b, the two siRNAs targeting YAP1 show somewhat diverging results where siYAP1 #7 resulted in a significant tamoxifen response already at treatment of 10^-7^ M 4-OH-tamoxifen, but siYAP1 #8 did not reach significance until a 10-fold higher tamoxifen concentration was used. Also, increasing concentrations of 4-OH-tamoxifen in siCtr-treated cells resulted in a more effective inhibition of cell viability. This effect could not be observed upon YAP1 depletion, in support of YAP1 being important for the 4-OH-tamoxifen effect. The general negative effect of YAP1 downregulation on cell viability somewhat complicates the interpretation of the result, however when measuring the specific activity of the estrogen response element upon E2 stimulation with or without YAP1, similar results were obtained. The fold decrease resulting from 4-OH-tamoxifen inhibition was less prominent when YAP1 was downregulated (siYAP1 3.33-3.79 vs. siCtr 4.52), indicating a role for YAP1 in mediating inhibition of ERE activity. The small but significant effect implies additional mechanisms contributing to YAP1’s role in tamoxifen resistance; for example, the known estrogen target gene cyclin D1 does not have an ERE promoter element, instead a cAMP response like element is suggested to be critical in mediating the transcriptional activation of the cyclin D1 gene upon E2 stimulation [64]. Possibly, measurement of cAMP response like element activation in a similar setting as described would yield additional support for the involvement of YAP1 in ER-mediated transcription.

## Conclusion

By analysing YAP1 mRNA and protein expression in a large number of primary breast tumours, we show that increased YAP1 is associated with more aggressive tumours in ER- breast tumours whereas in ER+ tumours, decreased YAP1 expression correlates to aggressiveness. These results clearly indicate the necessity of analysing ER+ and ER- breast tumours separately regarding YAP1 expression. Furthermore, low YAP1 mRNA expression was significantly associated with a decreased recurrence-free survival in the luminal A breast cancer subgroup, independently of lymph node status or proximal, possibly co-deleted 11q22 genes. This indicates a specific role for YAP1 in predicting outcome in this subgroup. Absent YAP1 protein expression was shown to be linked to an impaired tamoxifen response in a randomised patient material and *in vitro* experiments of YAP1 downregulation resulted in decreased sensitivity to tamoxifen, together with an increase in ER and PgR protein levels. Further research is warranted to elucidate the exact mechanism on YAP1 mediating endocrine resistance and its possible use as a marker in predicting response to tamoxifen.

## Abbreviations

YAP1: Yes-associated protein; ER: Estrogen receptor α; PgR: Progesterone receptor; ERE: Estrogen responsive element; ER+: Estrogen receptor α positive; ER-: Estrogen receptor α negative.

## Competing interests

The authors declare that they have no competing interests.

## Authors’ contributions

SL analysed YAP1 protein expression, performed statistical analyses of protein and gene expression data, designed and performed experiments and wrote the manuscript. NPT and AHS performed gene expression data analyses and critically revised the manuscript. KJ and OS participated in the design of the study, provided study material and information of patients, and revised the manuscript. HA designed experiments, analysed and interpreted data and critically revised the manuscript. GL participated in the design of the study, analysed and interpreted data, performed statistical analyses, analysed YAP1 protein expression and revised the manuscript. All authors read and approved the final manuscript.

## Pre-publication history

The pre-publication history for this paper can be accessed here:

http://www.biomedcentral.com/1471-2407/14/119/prepub

## Supplementary Material

Additional file 1 Correlations of YAP1 protein expression and clinical and molecular parameters of the ER- subgroup of the randomised cohort. Click here for file

Additional file 2 Correlations of YAP1 mRNA expression and clinical and molecular parameters of the ER- subgroup of the gene expression dataset. Click here for file

Additional file 3 ***CCND1 *** **amplification and *YAP1 *** **loss are inversely correlated on gene level.** Click here for file

Additional file 4 **Kaplan-Meier analysis of the screening cohort.** (a) YAP1 expression does not predict outcome in all patients (p = 0.342, log-rank test) or in (b) the subgroup of ER+ patients (p = 0.948, log-rank test). Click here for file

Additional file 5 **Cox multivariate regression analysis in the gene expression dataset.** YAP1 mRNA expression is an independent prognostic factor after adjustment of known prognostic factors. Click here for file

Additional file 6 YAP1 mRNA expression in breast cancer molecular subgroups of the gene expression dataset (n = 1107). Click here for file

Additional file 7 **Correlations of YAP1 mRNA and a selection of genes located at the same gene region as *YAP1*; 11q22.** Click here for file

Additional file 8 **Cox uni- and multivariate analysis based on the gene expression dataset and the genes selected from Additional file **7**.** Click here for file

Additional file 9 Growth curves of T47D and MCF-7 upon YAP1 downregulation and protein expression levels of YAP1 in breast cancer cell lines. Click here for file
