# Supplementary material for: Decreased expression of Yes-associated protein is associated with outcome in the luminal A breast cancer subgroup and with an impaired tamoxifen response
Source: BMC Cancer. 2014 Feb 22;14:119. doi: 10.1186/1471-2407-14-119 (PMC3937431; doi:10.1186/1471-2407-14-119)
Supplement: Additional file 1 — Correlations of YAP1 protein expression and clinical and molecular parameters of the ER- subgroup of the randomised cohort. [file 1471-2407-14-119-S1.pdf]

Additional file 1. Correlations of YAP1 protein expression and clinical and molecular parameters of the ER− subgroup of the randomised cohort.

	ER⁻ patients, n=151				
	YAP1 intensity, n=103				
	Absent n (%)	Weak n (%)	Intermediate n (%)	Strong n (%)	p-value
Variable	4 (4)	33 (32)	46 (45)	20 (19)	
NHG					
I	0	0	0	0	
II	1 (25)	6 (21)	4 (9)	1 (5)	
III	3 (75)	23 (79)	40 (91)	18 (95)	0.062[a]
Lymph node status					
Negative	1 (25)	10 (31)	13 (28)	10 (50)	
Positive	3 (75)	22 (69)	33 (72)	10 (50)	0.238[b]
Tumour size					
<20 mm	2 (50)	7 (21)	12 (26)	7 (35)	
≥20 mm	2 (50)	26 (79)	34 (74)	13 (65)	0.543[b]
PgR					
<10 %	4 (100)	29 (94)	42 (95)	20 (100)	
≥10 %	0	2 (6)	2 (5)	0	0.392[b]
Ki-67 fraction (%)					
0-10	1 (33)	11 (34)	6 (14)	1 (5)	
11-25	1 (33)	8 (25)	14 (32)	4 (21)	
26-100	1 (33)	13 (41)	24 (54)	14 (74)	0.005[a]
Cyclin D1 intensity					
absent/weak	4 (100)	28 (85)	41 (95)	20 (100)	
Intermediate/strong	0	5 (15)	2 (5)	0	0.060[b]
CCND1 amplification					
No	3 (100)	20 (95)	27 (100)	12 (100)	
Yes	0	1 (5)	0	0	0.291[b]

NHG=Nottingham histological grade, PgR=progesterone receptor
[a]Spearman´s rank correlation
[b]Mann-Whitney U test
