# Supplementary material for: Decreased expression of Yes-associated protein is associated with outcome in the luminal A breast cancer subgroup and with an impaired tamoxifen response
Source: BMC Cancer. 2014 Feb 22;14:119. doi: 10.1186/1471-2407-14-119 (PMC3937431; doi:10.1186/1471-2407-14-119)
Supplement: Additional file 2 — Correlations of YAP1 mRNA expression and clinical and molecular parameters of the ER- subgroup of the gene expression dataset. [file 1471-2407-14-119-S2.pdf]

Additional file 2. Correlations of YAP1 mRNA expression and clinical and molecular parameters of ER− subgroup of the gene expression dataset.

	ER− patients, n=239				
	YAP1 mRNA Quartiles				
	Q1	Q2	Q3	Q4	p-value
Variable	n=60	n=60	n=59	n=60	
NHG					
I	3 (7)	4 (10)	4 (11)	2 (5)	
II	19 (44)	13 (33)	12 (33)	15 (38)	
III	21 (49)	22 (57)	20 (56)	22 (57)	0.529[a]
Lymph node status					
Negative	49 (82)	52 (87)	48 (83)	53 (90)	
Positive	11 (18)	8 (13)	10 (17)	6 (10)	0.318[b]
Tumour size					
<20 mm	23 (52)	21 (51)	16 (42)	12 (31)	
≥20 mm	21 (48)	20 (49)	22 (58)	27 (69)	0.037 [b]
Cyclin D1 Quartiles					
Q1	19 (32)	13 (22)	11 (19)	17 (28)	
Q2	17 (28)	13 (22)	18 (31)	12 (20)	
Q3	7 (12)	17 (28)	15 (25)	20 (33)	
Q4	17 (28)	17 (28)	15 (25)	11 (19)	0.884 [a]

NHG=Nottingham histological grade
[a]Spearman´s rank correlation
[b]Mann-Whitney U test
