# Supplementary material for: Decreased expression of Yes-associated protein is associated with outcome in the luminal A breast cancer subgroup and with an impaired tamoxifen response
Source: BMC Cancer. 2014 Feb 22;14:119. doi: 10.1186/1471-2407-14-119 (PMC3937431; doi:10.1186/1471-2407-14-119)
Supplement: Additional file 3 — CCND1 amplification and YAP1 loss are inversely correlated on gene level. [file 1471-2407-14-119-S3.pdf]

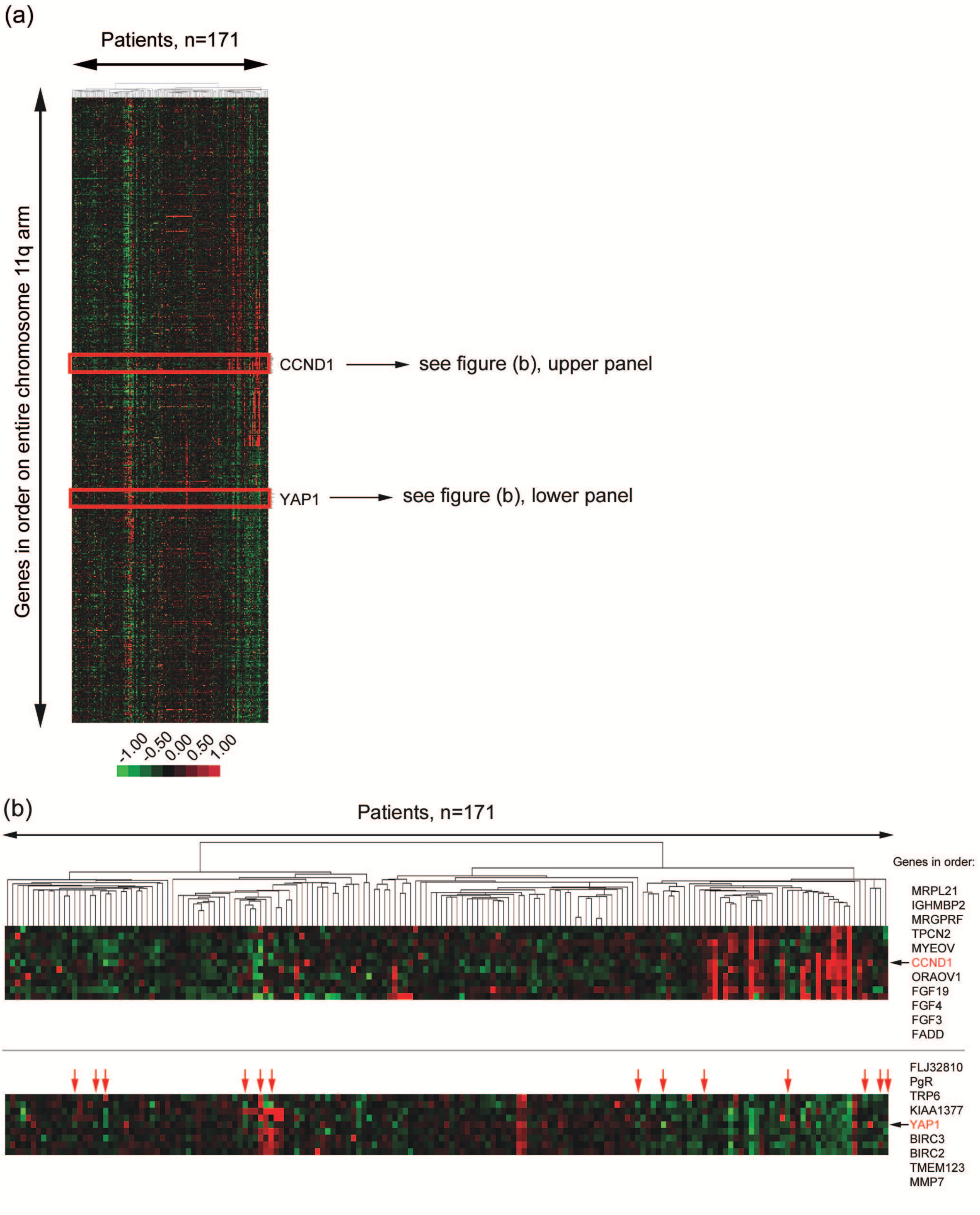

Additional file 3.pdf *CCND1* amplification and *YAP1* loss are inversely correlated on gene level (a) aCGH data of 171 patients was subjected to semi-supervised hierarchical clustering analysis and shown here is a heatmap of genes of the entire chromosomal 11q arm in order. (b) Selected regions from the heatmap in (a) shows that amplification of *CCND1* and proximal genes correlate to chromosomal loss of *YAP1* region. Red arrows indicate tumours which show loss of *YAP1* but no concurrent *CCND1* amplification is detected.
aCGH=array Comparative Genomic Hybridisation
