# Supplementary material for: Decreased expression of Yes-associated protein is associated with outcome in the luminal A breast cancer subgroup and with an impaired tamoxifen response
Source: BMC Cancer. 2014 Feb 22;14:119. doi: 10.1186/1471-2407-14-119 (PMC3937431; doi:10.1186/1471-2407-14-119)
Supplement: Additional file 4 — Kaplan-Meier analysis of the screening cohort. (a) YAP1 expression does not predict outcome in all patients (p = 0.342, log-rank test) or in (b) the subgroup of ER+ patients (p = 0.948, log-rank test). [file 1471-2407-14-119-S4.pdf]

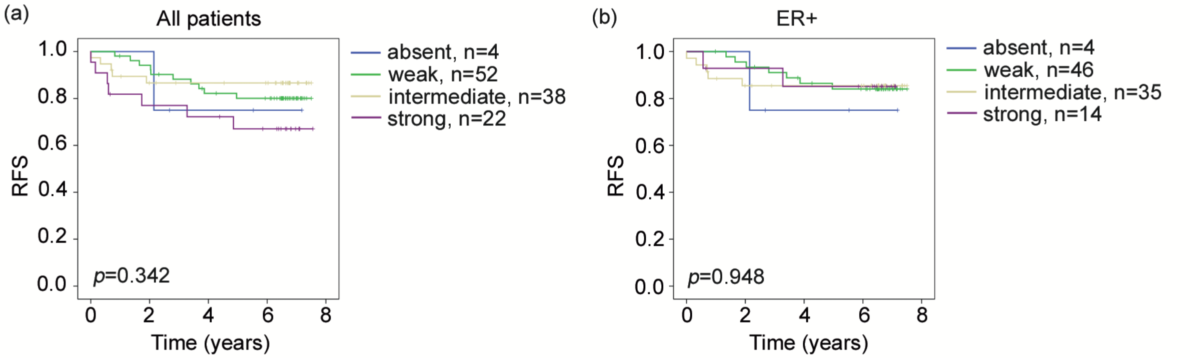

Additional file 4.Kaplan-Meier analysis of the screening cohort. **(a)** YAP1 expression does not predict outcome in all patients (log-rank test) or in **(b)** the subgroup of ER+ patients.
