# Supplementary material for: Decreased expression of Yes-associated protein is associated with outcome in the luminal A breast cancer subgroup and with an impaired tamoxifen response
Source: BMC Cancer. 2014 Feb 22;14:119. doi: 10.1186/1471-2407-14-119 (PMC3937431; doi:10.1186/1471-2407-14-119)
Supplement: Additional file 5 — Cox multivariate regression analysis in the gene expression dataset. YAP1 mRNA expression is an independent prognostic factor after adjustment of known prognostic factors. [file 1471-2407-14-119-S5.pdf]

**Additional file 5. Cox multivariate regression analysis in the gene expression dataset (n=1107)**. YAP1 mRNA expression is an independent prognostic factor after adjustment of known prognostic factors.

| | Univariate | | | Multivariate | | |
|---|---|---|---|---|---|---|
| Variable | HR | 95% CI | *P* value | HR | 95% CI | *P* value |
| Grade[†] | 2.307 | 1.611-3.302 | <0.001 | 1.978 | 1.349-2.902 | <0.001 |
| Tumour size[‡] | 1.533 | 1.196-1.964 | 0.001 | 1.399 | 1.075-1.819 | 0.012 |
| Lymph node[¦] | 1.284 | 0.972-1.697 | 0.078 | 1.209 | 0.894-1.634 | 0.217 |
| YAP1[*] | 1.345 | 1.104-1.639 | 0.003 | 1.306 | 1.014-1.683 | 0.039 |

[†] Nottingham histological grade I vs. II and III
[‡] Size ≤20 vs. >20 mm
[¦] Lymph node, negative vs. positive
[*] High vs. low mRNA expression
HR=Hazard ratio, CI=Confidence interval
