# Supplementary material for: Decreased expression of Yes-associated protein is associated with outcome in the luminal A breast cancer subgroup and with an impaired tamoxifen response
Source: BMC Cancer. 2014 Feb 22;14:119. doi: 10.1186/1471-2407-14-119 (PMC3937431; doi:10.1186/1471-2407-14-119)
Supplement: Additional file 6 — YAP1 mRNA expression in breast cancer molecular subgroups of the gene expression dataset (n = 1107). [file 1471-2407-14-119-S6.pdf]

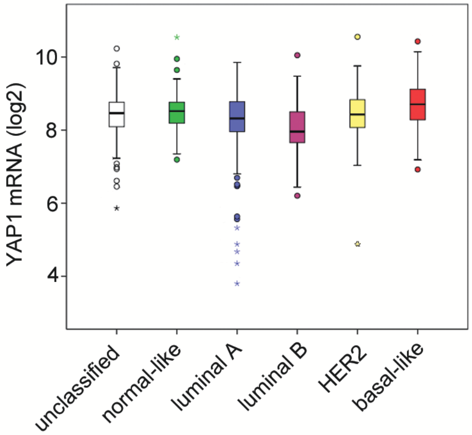

Additional file 6. A box plot showing the interquartile range of YAP1 mRNA in breast cancer molecular subgroups of the gene expression dataset (n=1107).
Luminal A and B have significantly lower mRNA expression compared to normal- and basal-like (Kruskal-Wallis Post Hoc test, not assuming equal variances, $p<0.001$). Luminal B tumours also have lower mRNA expression compared to the HER2 subgroup ($p<0.001$). There is no statistical difference regarding YAP1 expression in luminal A and B subgroups ($p=0.185$).
