# Supplementary material for: Decreased expression of Yes-associated protein is associated with outcome in the luminal A breast cancer subgroup and with an impaired tamoxifen response
Source: BMC Cancer. 2014 Feb 22;14:119. doi: 10.1186/1471-2407-14-119 (PMC3937431; doi:10.1186/1471-2407-14-119)
Supplement: Additional file 7 — Correlations of YAP1 mRNA and a selection of genes located at the same gene region as YAP1 ; 11q22. [file 1471-2407-14-119-S7.pdf]

Additional file 7. Correlations of YAP1 mRNA and a selection of genes (in order) located at the same gene region as *YAP1*; 11q22. An arbitrary cut-off of Spearman's rho was set at 0.2 since higher correlations coefficients would be more likely to indicate co-deletion with YAP1. Genes chosen for further analysis are highlighted in bold.

	All subtypes n=1107		Luminal A subtype n=286	
Gene selection of 11q22 (in order) mRNA vs. YAP1 mRNA	Spearman's rho	P value	Spearman's rho	P value
CNTN5	0.020	0.507	-0.051	0.386
PgR	-0.003	0.921	0.150	0.011
YAP1	1.00	-	1.00	-
BIRC3	0.065	0.030	0.182	0.002
BIRC2	**0.399**	**<0.001**	**0.426**	**<0.001**
TMEM123	**0.445**	**<0.001**	**0.442**	**<0.001**
MMP7	**0.227**	**<0.001**	0.042	0.475
MMP20	0.054	0.070	0.053	0.376
MMP27	-0.005	0.873	-0.089	0.133
MMP13	0.051	0.088	0.036	0.548
