# Supplementary material for: Decreased expression of Yes-associated protein is associated with outcome in the luminal A breast cancer subgroup and with an impaired tamoxifen response
Source: BMC Cancer. 2014 Feb 22;14:119. doi: 10.1186/1471-2407-14-119 (PMC3937431; doi:10.1186/1471-2407-14-119)
Supplement: Additional file 8 — Cox uni- and multivariate analysis based on the gene expression dataset and the genes selected from Additional file 7. [file 1471-2407-14-119-S8.pdf]

Additional file 8. Cox uni- and multivariate analysis based on the gene expression dataset including genes correlating with YAP1 mRNA expression. In the entire dataset, only grade and tumour size are significant in the multivariate analysis. In the luminal A subgroup, YAP1 mRNA is the only significant factor remaining after adjusting for additional 11q22 genes. Cyclin D1 was included in the analysis due to its inverse correlation to YAP1.

	All subtypes, n=1107					
	Univariate			Multivariate		
Variable	HR	95% CI	*P* value	HR	95% CI	*P* value
Grade[†]	2.31	1.61 to 3.30	<0.001	1.98	1.35 to 2.92	0.001
Tumour size[‡]	1.53	1.20 to 1.96	0.001	1.38	1.06 to 1.79	0.018
Lymph node[¦]	1.28	0.97 to 1.70	0.078	1.21	0.89 to 1.63	0.225
YAP1*	1.35	1.10 to 1.64	0.003	1.24	0.94 to 1.63	0.125
BIRC2*	1.32	1.09 to 1.61	0.006	1.20	0.91 to 1.59	0.206
TMEM123*	1.15	0.94 to 1.40	0.169	1.10	0.82 to 1.48	0.511
MMP7*	0.90	0.74 to 1.10	0.274	0.88	0.67 to 1.14	0.325
	Luminal A, n=286					
	Univariate			Multivariate		
Variable	HR	95% CI	*P* value	HR	95% CI	*P* value
Grade[†]	1.56	0.86 to 2.81	0.144	1.37	0.72 to 2.61	0.343
Tumour size[‡]	1.26	0.75 to 2.10	0.386	1.09	0.62 to 1.92	0.769
Lymph node[¦]	1.86	1.10 to 3.14	0.021	1.77	0.96 to 3.26	0.067
YAP1*	2.34	1.51 to 3.62	<0.001	1.99	1.12 to 3.56	0.019
BIRC2*	1.41	0.92 to 2.14	0.114	1.01	0.56 to 1.81	0.975
TMEM123*	1.73	1.13 to 2.65	0.012	1.71	0.92 to 3.19	0.088
CCND1*	0.60	0.39 to 0.91	0.017	0.65	0.37 to 1.13	0.130

HR=Hazard ratio, CI=Confidence Interval
[†] Nottingham histological grade I vs. II and III
[‡]Size ≤20 vs. >20 mm
[¦]Lymph node, negative vs. positive
[*]High vs. low mRNA levels
