# Supplementary material for: Decreased expression of Yes-associated protein is associated with outcome in the luminal A breast cancer subgroup and with an impaired tamoxifen response
Source: BMC Cancer. 2014 Feb 22;14:119. doi: 10.1186/1471-2407-14-119 (PMC3937431; doi:10.1186/1471-2407-14-119)
Supplement: Additional file 9 — Growth curves of T47D and MCF-7 upon YAP1 downregulation and protein expression levels of YAP1 in breast cancer cell lines. [file 1471-2407-14-119-S9.pdf]

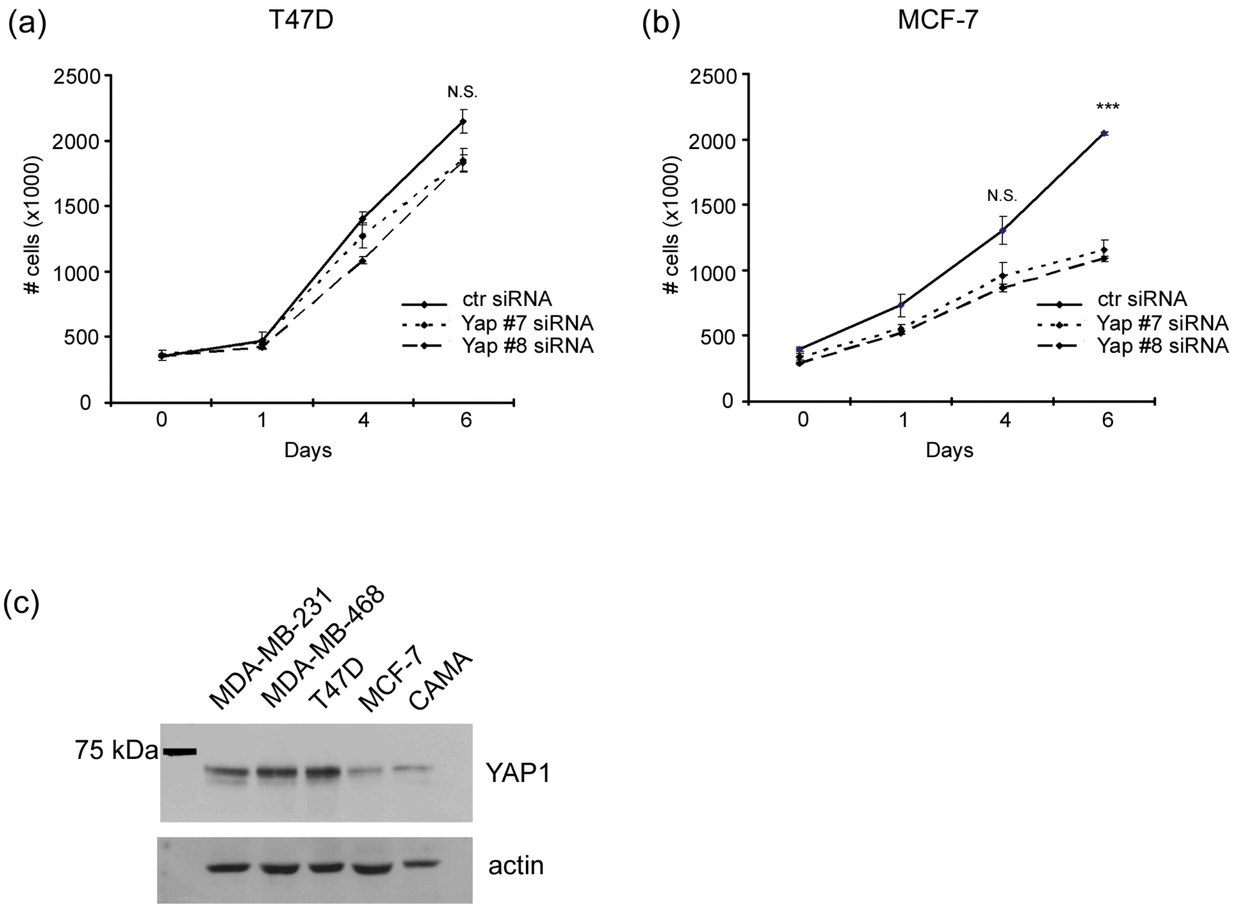

Additional file 9. (a-b) T47D and MCF-7 cells were seeded in duplicate in 6-well plates, transfected with ctr, Yap #7 or Yap #8 siRNA and counted at the indicated time-points. T47D cells were grown in full DMEM and MCF-7 cells in full RPMI medium. Day 0 denotes 48 hours after transfection. (c) Western blot of YAP1 protein expression in breast cancer cell lines. ER+ cell lines: T47D, MCF-7 and CAMA. ER- cell lines: MDA-MB-231 and MDA-MB-468.
